# P-452. Factors Associated with Liver Fibrosis Development in HIV-Monoinfected Patients Within the Modern ART Era

**DOI:** 10.1093/ofid/ofae631.652

**Published:** 2025-01-29

**Authors:** Morgan L Endreson, Hsing-Chuan Hsieh, Christie Joya, Rhonda E Colombo, Christina Schofield, Tahaniyat Lalani, Derek Larson, Joseph Yabes, Robert O’Connell, Jason Blaylock, Brian Agan, Anuradha Ganesan

**Affiliations:** National Capital Consortium, College Park, Maryland; Infectious Disease Clinical Research Program, Bethesda, Maryland; Naval Medical Center Portsmouth, Portsmouth, Virginia; Infectious Disease Clinical Research Program, USUHS; Henry M. Jackson Foundation for the Advancement of Military Medicine, Inc., Bethesda, Maryland; Madigan Army Medical Center, Tacoma, Washington; Naval Medical Center Portsmouth, Portsmouth, Virginia; Fort Belvoir Community Hospital and Uniformed Services University, Fort Belvoir, Virginia; Brooke Army Medical Center, San Antonio, Texas; Infectious Disease Clinical Research Program, USUHS, Bethesda, Maryland; Walter Reed National Military Medical Center, Bethesda, Maryland; Infectious Disease Clinical Research Program, Department of Preventive Medicine and Biostatistics, Uniformed Services University of the Health Sciences, Bethesda, MD, USA, Bethesda, Maryland; Infectious Disease Clinical Research Program, USUHS; Henry M. Jackson Foundation for the Advancement of Military Medicine Inc, Bethesda, Maryland

## Abstract

**Background:**

Liver fibrosis (LF) is a significant contributor to morbidity and mortality in people living with HIV (PLWH). Using data from the US Military HIV Natural History Study (NHS), we examined prevalence and risk factors of LF in the era of fixed dose combination antiretroviral therapy (ART).

**Table 1.**
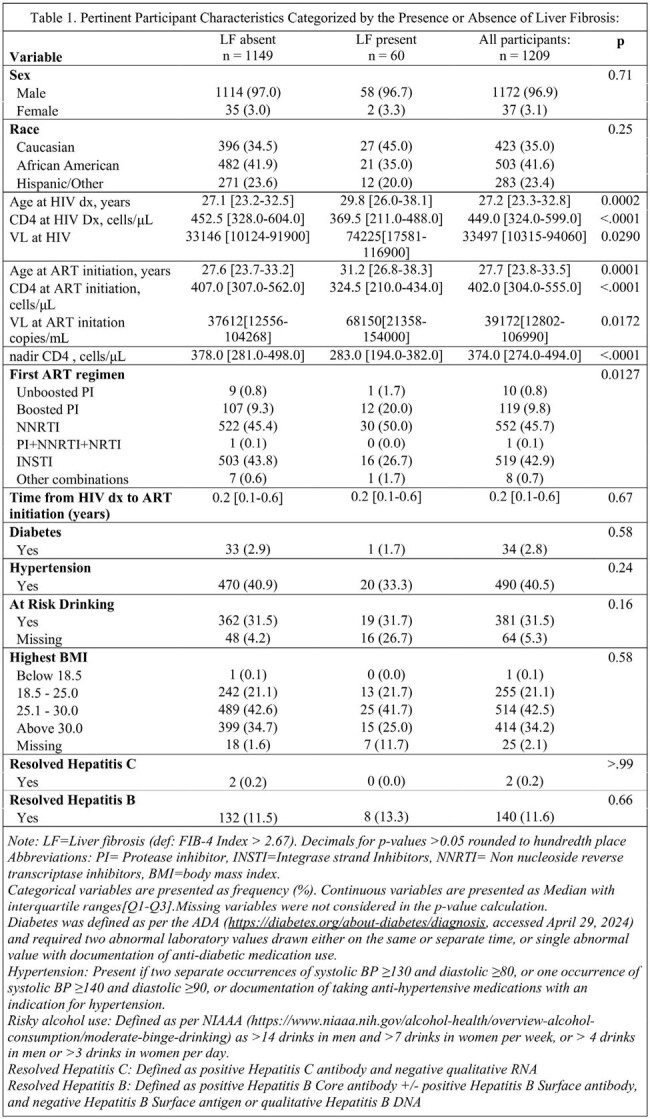
Pertinent Participant Characteristics Categorized by the Presence or Absence of Liver Fibrosis:

**Methods:**

The NHS is comprised of military beneficiaries with HIV, who are often diagnosed and treated early due to mandatory screening and guideline-based initiation of ART. We included ART-treated NHS participants, diagnosed after 2006, without active hepatitis B or C infection. LF was defined as a FIB-4 Index >2.67, a validated, non-proprietary, readily available marker of LF based on age, platelets, and liver-associated enzymes. Risk factors examined included demographic, HIV-specific, and other recognized associations with LF, see Table 1 footnote for details. Participants characteristics were analyzed with either the Kruskal Wallis or Chi2 test. Cox proportional hazard models, adjusted for baseline and time-updated variables, were used to assess risk factors for developing LF.

**Table 2.**
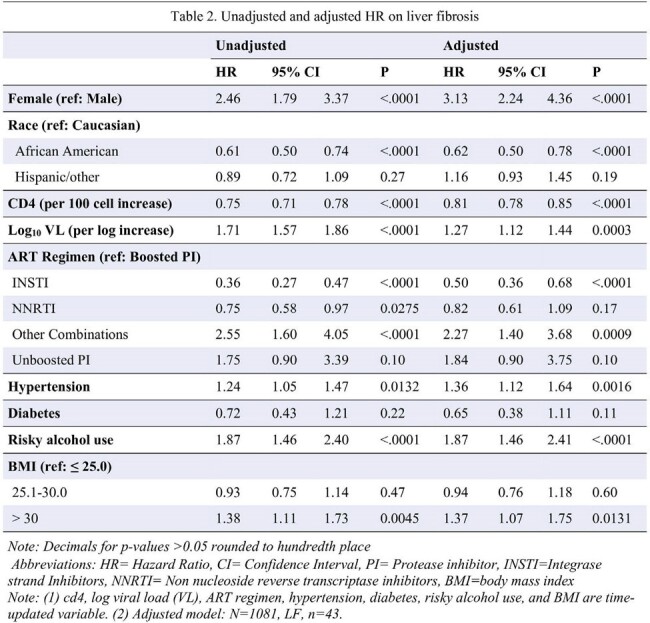
Unadjusted and adjusted HR on liver fibrosis

**Results:**

Among 1209 participants, followed for a median of 5.4 years, 60 (5.0%) met the criteria for LF. Overall, the median time to ART initiation was 2.4 months. The median time to LF was 2.5 years. At LF diagnosis the median age, CD4 count and viral load were 36 years, 526 cells/uL and 20 copies/mL respectively. Those with LF were older, had lower CD4 count and high VL at HIV diagnosis and more likely to initiate NNRTI based ART, table 1. In an adjusted model, LF was associated with female gender, hypertension, obesity, at risk drinking, and higher VL, while African American race, integrase strand transfer inhibitor (INSTI) use, and higher CD4 counts were protective, table 2.

**Conclusion:**

In the contemporary ART era, in this young and promptly treated cohort, the prevalence of LF was 5%, suggesting liver-related morbidity remains an important concern. Our results underscore the protective effect of immune preservation (by timely initiation and continuation of ART) against LF; however, the observed protective association with INSTI-based regimens will need further study to determine long-term benefit. As observed previously, hypertension, at risk drinking, and obesity are modifiable risk factors that should be addressed.

**Disclosures:**

**All Authors**: No reported disclosures

